# The Genome Survey Analysis of Female and Male *Sepiella japonica*

**DOI:** 10.3390/genes16101215

**Published:** 2025-10-15

**Authors:** Yuting Ren, Yinquan Qu, Fenglin Wang, Tianxiang Gao, Xiumei Zhang

**Affiliations:** 1 School of Marine Sciences, Ningbo University, Ningbo 315211, China; 2301130093@nbu.edu.cn (Y.R.); wfl941122@126.com (F.W.); 2 Fishery College, Zhejiang Ocean University, Zhoushan 316022, China; qyquan@njfu.edu.cn (Y.Q.); gaotianxiang0611@163.com (T.G.)

**Keywords:** *Sepiella japonica*, genome survey, genomic characteristics

## Abstract

Background/Objectives: *Sepiella japonica* is a highly adaptable cephalopod with an advanced nervous system and complex reproductive behavior, capable of reproducing two to three generations annually depending on water temperature. However, the absence of a complete genome assembly has limited molecular investigations of its unique biological characteristics. This study aimed to perform a genome survey of female and male *S. japonica*, systematically characterize and compare key genomic characteristics. Methods: Quality-filtered short reads enabled K-mer-based estimation of genome size, heterozygosity, repeat content, and GC content; generation of draft genome assemblies, SSR identification from the draft assemblies, complete mitogenome assemblies and annotations with ML phylogeny based on 13 concatenated PCGs, and PSMC-based demographic inference. Results: The estimated genome sizes were 4317 Mb (female) and 4222 Mb (male), with revised estimates of 4310 Mb and 4215 Mb, respectively. K-mer analysis revealed heterozygosity rates of 0.85% (female) and 0.77% (male) and repeat content of 76.05% (female) and 75.91% (male). The assembled genome sizes were 4197 Mb for females (N50: 508 bp) and 4206 Mb for males (N50: 511 bp); the GC content was 34.15% for both genomes. Deduplicated data showed GC content of 35.16% (female) and 35.27% (male). Microsatellite analysis revealed that mononucleotide repeats were the most abundant simple sequence repeat motif. The mitochondrial genome sequences measured 16,729 bp for the female genome and 16,725 bp for the male genome. Conclusions: This study provides fundamental data for subsequent high-quality whole-genome assembly and comparative analysis of female and male genomes.

## 1. Introduction

*Sepiella japonica* is a mollusk in the class Cephalopoda (Sepiida: Sepiidae) [1]. It is one of the four traditional marine species in the East China Sea and possesses notable economic and medicinal value. This species is widely distributed in the coastal waters of the Northwest Pacific and the Northern Indian Ocean, with particularly large populations occurring in China’s Liaodong Bay, Zhoushan Archipelago, and Beibu Gulf. Populations of *S. japonica* declined severely during the 1980s due to overfishing and environmental degradation. This species can grow rapidly, has a short life cycle, and migrates to reproduce [2]. In recent years, stocking strategies, including artificial breeding and release, have been optimized, and this has greatly aided population recovery. However, the effectiveness of these programs has been limited by various challenges such as precocious maturation and miniaturization of individuals. These phenotypic abnormalities are likely associated with the species’ reproductive biology and mechanisms regulating sex differentiation, but the molecular basis of these mechanisms has not yet been clarified. The lack of whole-genome data and systematic female and male genomic comparisons has constrained the elucidation of these molecular mechanisms.

Previous genomic studies on cephalopods have revealed that representative species generally possess large genome sizes and high proportions of repetitive elements. For example, the genome of *Sepia officinalis* is estimated at 5 Gb, with 71.17% of bases masked as repetitive elements. The first sequenced cephalopod genome, *Octopus bimaculoides* (2.7 Gb), exhibits extensive gene family expansions and large-scale genome rearrangements [3]. The chromosome-scale genome assembly of *Octopus vulgaris* revealed a genome size of 2.8 Gb, with repeats comprising 68.68%, thereby advancing comparative research on genome structure and karyotype evolution [4]. The genome of *Sepia pharaonis* (4.79 Gb, 65% repeats) uncovered a unique reflectin gene family associated with body coloration, including 12 reflectin copies and three newly classified types [5]. The genome (5.1 Gb) and transcriptome resources of *Euprymna scolopes* provided genomic insights into host–microbe interactions and the evolution of symbiotic organs [6]. In contrast, the genome of *Nautilus pompilius* (730 Mb) is markedly smaller than that of other cephalopods, with TEs making up about 31% dominated by DNA transposons, and displays a pronounced contraction of orthologous gene families rather than expansion. This assembly provides a novel perspective on the evolution of cephalopod vision and biomineralization [7]. Review studies have emphasized that the extraordinary complexity of cephalopod genomes arises mainly from mechanisms such as gene family expansion, widespread RNA editing, large-scale rearrangements, and repeat-mediated regulatory innovations [8,9,10]. Together, these processes underlie unique traits such as advanced camouflage and neural plasticity. Overall, these studies indicate that cephalopod genomes are generally large, rich in repetitive sequences, and evolutionarily dynamic, which likely reflect the selective pressures imposed by rapid growth, short life cycles, and complex reproductive strategies. These results highlight the importance of investigating female and male genomic characteristics and their differences to better understand the adaptive evolution of cephalopods.

Whole-genome sequencing has become a key tool in contemporary biological research [11], and DNA Nanoball (DNB) sequencing has been widely applied in various genomic studies [12]. This technology allows analysis of basic genomic features such as genome size, GC content, and heterozygosity [13,14,15,16,17,18,19], and enables identification of mitochondrial genomes, microsatellite markers, and single-copy orthologous genes [20,21,22,23,24,25]. Characterizing genomic differences between males and females is crucial for elucidating the molecular mechanisms of sex determination and differentiation and provides key clues for understanding reproductive biology and adaptive evolution. In recent years, several studies have focused on sex-related genomic differences in mollusks. For example, Kina et al. compared female and male *Haliotis gigantea* by whole-genome resequencing, revealing about 2 Mb of sex-related genomic differences on chromosome 18, clarifying its XX/XY genetic sex-determination system, and developing male-specific molecular markers [26]. Similarly, Zou et al. identified sex-linked SNP and InDel loci in resequencing data ivory shell (*Babylonia areolata*), aiding the elucidation of its sex-determination system [27]. However, comparative analyses of female and male genomes in cephalopods remain limited, and studies on *S. japonica* are particularly lacking. Given the unique reproductive strategies and evolutionary pressures of cephalopods, their mechanisms of sex differentiation are closely related to adaptive evolution, making comparative genomic analysis particularly important for revealing the adaptive evolution of *S. japonica*.

In this study, female and male *S. japonica* genomes were sequenced using next-generation sequencing (NGS) technology. The K-mer method was used to estimate key genomic parameters, including genome size, heterozygosity, GC content, repeat content, and genome integrity, thereby describing the genomic characteristics of female and male individuals. In addition, the distribution of simple sequence repeats (SSRs) was analyzed, and the mitochondrial genome was annotated. Our findings will provide foundational data for comparative genomic analyses between males and females and will offer a basis for future studies on the genetic basis and evolutionary mechanisms of sex determination and differentiation in cephalopods, as well as inform molecular breeding and germplasm resource conservation.

## 2. Materials and Methods

### 2.1. Ethics Statement

The samples used in this study were artificially cultured and collected postmortem. All *S. japonica* specimens were processed following procedures that were compliant with the Animal Care and Use Ethics policies of Zhejiang Ocean University (Approval No. 2024150).

### 2.2. Sample Collection and Genome Sequencing

Six *S. japonica* samples (three males and three females) were collected from the Xishan Island in May 2024. The muscle tissue obtained through dissection was preserved at −80 °C. About 1 g of muscle tissue was collected for DNA extraction. DNA was extracted using the phenol/chloroform method. The concentration, purity, and integrity of DNA were evaluated using a NanoDrop 2000 (Thermo Fisher Scientific Inc., Waltham, MA, USA) and 1% agarose gel electrophoresis. Two paired-end DNA libraries with an insert size of 350 bp were constructed and sequenced on the DNBseq platform (BGI) according to the manufacturer’s protocol. Library construction and sequencing were performed by OneMore Technology Co., Ltd. (Wuhan, China).

### 2.3. K-mer Analysis and Genome Assembly

Quality control and preprocessing of the raw sequencing data were performed using FastQC (v0.11.3) [28] and FASTP (v0.23.2) [29]. Filtering of the raw data was conducted with FASTP, with the length parameter “-l 50” and using default settings for other parameters. Initial sequencing quality was evaluated with FastQC using default parameters. Sequencing quality was evaluated using various quality metrics, including Q20 (the proportion of bases with a Phred quality score greater than 20), Q30 (the proportion of bases with scores greater than 30), and the GC content distribution. BLAST (2.11.0+) was used to align a random subset of 10,000 high-quality read pairs selected from the filtered data to the NCBI nucleotide (NT) database. Matches in the top 80% were identified and visualized for downstream analysis [30]. Rigorous quality control and filtering were performed (trimming low-quality bases [Q < 20], removing adapter sequences, discarding reads with >40% low-quality bases [Q < 15] or >5 ambiguous bases [N], retaining reads ≥50 bp, and eliminating PCR duplicates), which yielded high-quality clean reads for subsequent analyses. K-mer analysis was performed using clean reads in Jellyfish software (version 2.3.0, parameters: -m 21 -s 1e9 -p 2), and K-mer frequency statistics were calculated [31]. The peak depth and optimal K-mer quantity were determined based on the K-mer analysis results. The following formula was used to estimate the genome size: Genome size (G) = n_k-mer_/C_k-mer_ = N_base_/C_base_, where n_base_ and n_K-mer_ represent the total number of bases and the number of K-mers in the sequence, respectively, and C_base_ and C_K-mer_ denote the average depth of base pairs and K-mer coverage, respectively. The heterozygosity and repeat sequence ratio of the genome were also evaluated using K-mer analysis [32]. GCE (v1.0.0) was used to perform K-mer analysis with a K-mer size of 17, which yielded up to 4^17^ possible K-mer types; this was sufficient for ensuring comprehensive genomic coverage [33]. The short-read assembler Minia was used to assemble clean reads with the de Bruijn graph algorithm using a k-mer size of 51 [34]. PRINSEQ (https://sourceforge.net/projects/prinseq/ (accessed on 20 March 2025)) was used with the -stats all parameter to evaluate the assembled sequences, and BBMap (v39.13) was used to calculate the GC content. Smudgeplot (v0.2.3dev) was used to visualize heterozygous K-mer pair patterns to further characterize genome structure and ploidy.

### 2.4. Microsatellite Identification

Microsatellite motifs in the draft genome were identified using the MIcroSAtellite tool (http://pgrc.ipk-gatersleben.de/misa/ (accessed on 25 March 2025)). The analysis was performed using standard parameters: the minimum number of repeats was set to 6, 5, 5, 6, and 5 for di-, tri-, tetra-, penta-, and hexanucleotide motifs, respectively.

### 2.5. Mitochondrial Genome Assembly and Phylogenetic Analysis

GetOrganelle (v1.7), Novoplasty (v4.3.5), and MitoZ (v3.6) were used to assemble the filtered reads into a complete mitochondrial genome. For MitoZ, default parameters were applied except for specifying Mollusca as the clade, genetic code 33, and species name *S. japonica*. GetOrganelle was run with default parameters. NOVOPlasty was executed with the recommended configuration file, in which the seed sequence and species information were customized. A reference sequence from the closely related species *Sepia esculenta* (accession number AB266516.1) was downloaded from NCBI (https://www.ncbi.nlm.nih.gov/, accessed on 8 August 2024). SeqMan Ultra was used to refine the assembled sequence to ensure its completeness. The MITOS Web Server [35] was used to annotate the mitochondrial genomes, and OGDRAW (https://chlorobox.mpimp-golm.mpg.de/OGDraw.html (accessed on 23 August 2025)) was used to generate circular genome maps. The 13 protein-coding genes (PCGs) were successfully identified in this study.

The mitochondrial genomes of 17 cephalopod species were analyzed, including seven representatives from Sepiidae (*Sepia*, *Metasepia*, *Sepiella)* and outgroup taxa (*Nautilus*, *Octopus*, *Todarodes*), with *S. japonica* included as the focal species in this study (Table 1). Thirteen protein-coding genes (PCGs: *atp6*, *atp8*, *cox1-3*, *cob*, *nad1-6*, *nad4l*) were extracted for phylogenetic analysis. Nucleotide sequences of each PCG were initially aligned using MAFFT (v7.505) [36]. The resulting alignments were subsequently refined using TrimAl (v1.4.1) [37]. The trimmed alignments of all 13 PCGs were then concatenated into a supermatrix using PhyloSuite (v1.2.3) [38]. Phylogenetic reconstruction was performed using maximum likelihood in IQ-TREE (v2.2.0), with the best-fit substitution model (MFP) automatically selected by ModelFinder [39]. The analysis incorporated 1000 ultrafast bootstrap replicates for branch support estimation. The resulting phylogenetic tree was rooted with *N. pompilius* (Nautiloidea) as the outgroup and visualized using FigTree (v1.4.4) and iTOL for further examination of topological relationships within Sepiidae and related coleoid lineages [40].

### 2.6. Effective Population Size Inferrence

The Pairwise Sequentially Markovian Coalescent (PSMC) method was used to infer the historical population dynamics of *S. japonica*. The PSMC model estimates changes in effective population size over time by analyzing the distribution of heterozygous sites across the genome of a single diploid individual [41]. Paired-end sequencing reads were first aligned to the reference genome using BWA-mem. The resulting “sam” files were sorted into “bam” format with Samtools (sort, -@ 8). High-quality “consensus.fq” files were then generated with bcftools and vcftools, and converted to psmcfa format using the PSMC script “fq2psmcfa” (-q20) [42]. PSMC was subsequently run with the generation interval (g) of 0.5 years and the mutation rate (µ) of 0.9 × 10^−8^, following estimates for the mollusk *Haliotis sorenseni* [43].

## 3. Results

### 3.1. Size, Heterozygosity Ratio, and Repeat Sequence Ratio

**Figure 1:**
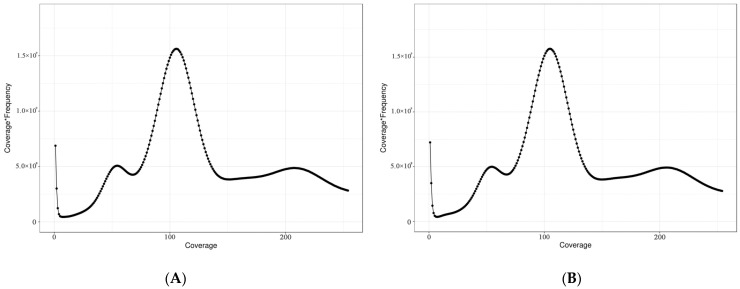
Distribution of the 17-mer depth and frequency in the female and male *S. japonica* genomes. (**A**) Female. (**B**) Male. The *x*-axis (Coverage) represents the sequencing depth; the *y*-axis represents the proportion of reads at each depth relative to the total read count.

A total of 516.85 Gb and 518.63 Gb of raw sequencing data were obtained for female and male *S. japonica*, respectively. A total of 488.58 Gb and 488.46 Gb of high-quality clean reads were obtained after quality filtering and removing redundant reads, respectively. The Q20 and Q30 values of all six libraries were greater than 99.40% and 97.83%, respectively, indicating that the quality of the sequencing data was high. The average GC content was 35.16% for females and 35.27% for males (Table 2). The sequencing depth was 102× for both female and male *S. japonica* according to K-mer analysis (Figure 1); the estimated genome sizes were 4317 Mb and 4222 Mb, and the revised genome sizes were 4310 Mb and 4215 Mb for females and males, respectively. Heterozygosity rates were 0.85% in females and 0.77% in males, and repeats comprised 76.05% and 75.91% of the female and male genomes, respectively (Table 3). No exogenous contamination was detected in the sequencing data according to the alignment of clean reads against the NCBI NT database (Table S1).

**Figure 2:**
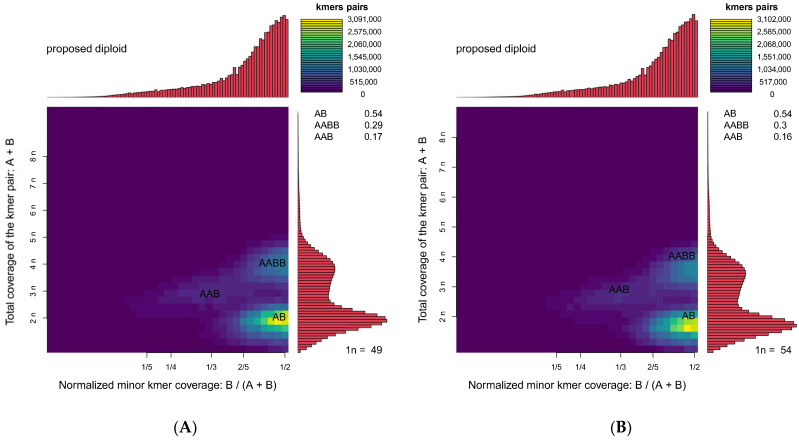
Genome ploidy level analysis of female and male *S. japonica*. (**A**) Female. (**B**) Male. The plots display the heterozygous K-mer distribution for diploid *S. japonica*. The concentration of points in defined regions is consistent with the features expected for a diploid genome.

The draft genome assembly was generated using filtered clean reads. Information on the draft genome for female and male *S. japonica* at the contig level is shown in Table 4. Mapping rates of 99.78% (female) and 99.77% (male) based on 10 Gb subsampled reads confirmed the high completeness and low contamination of the draft assemblies. The total contig length for the female genome was 4197 Mb, which comprised 21,378,004 contigs, with N50 and N90 lengths of 508 bp and 57 bp, respectively, and a maximum contig length of 23,523 bp. The GC content of the assembled female genome was 34.15%. The total contig length of the male genome was 4206 Mb, which comprised 21,355,316 contigs, with N50 and N90 lengths of 511 bp and 57 bp, respectively, and the maximum contig length was 23,780 bp. The total length and N50 length were greater for the male genome than the female genome; the number of contigs was slightly lower in the male genome than in the female genome. Smudgeplot analysis revealed that *S. japonica* is a diploid species (AB) (Figure 2, Table 5), and AB-type K-mers were the most common (54%). The presence of AABB and AAB peaks suggests a high repeat content and heterozygosity rates [44,45]. These values suggest that levels of heterozygosity were similar in males and females, which indicates that the architecture of the genome is conserved across sexes; sex-linked chromosomal divergence was absent.

### 3.2. Identification of Microsatellite Motifs

**Figure 3:**
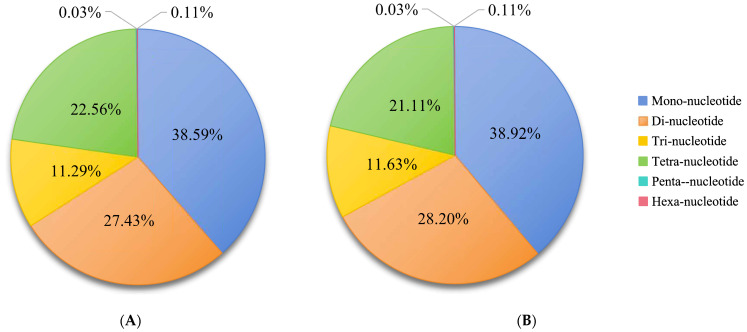
Frequency of SSR types in the genomic survey of female and male *S. japonica*. (**A**) Female. (**B**) Male.

**Figure 4:**
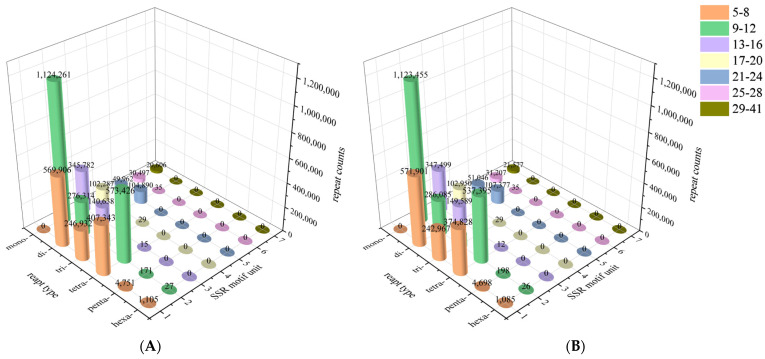
Distribution of microsatellite repeats in female and male *S. japonica*. (**A**) Female. (**B**) Male.

Both ends of the contig sequences from the assembled draft genome sequences were screened to identify SSR sequences. A total of 21,355,316 and 21,378,004 microsatellite motifs were identified in female and male *S. japonica* genomes, respectively (Table 6). In the male genome, microsatellite motifs were 38.92% mononucleotide repeats, 28.20% dinucleotide repeats, 11.63% trinucleotide repeats, 21.00% tetranucleotide repeats, 0.11% pentanucleotide repeats, and only a few hexanucleotide repeats. By contrast, microsatellite motifs in the female genome were 38.59%, 27.43%, 11.29%, 21.00%, and 0.11% mono-, di-, tri-, tetra-, penta-, respectively (Figure 3). Overall, the microsatellite motif profiles of female and male *S. japonica* genomes were highly similar (Figure 3 and Figure 4; Table 6). The numbers of most repeats for *S. japonica* ranged from 9 to 12 (mono-) and 5 to 8 (di-, tri-, tetra-) for both males and females.

### 3.3. Characterization of S. japonica Mitochondrial Genome

The complete mitochondrial genomes of female and male *S. japonica* formed closed circular molecules, with total lengths of 16,729 bp and 16,725 bp, respectively. These two mitochondrial genomes comprised 37 genes, including 13 PCGs (7 NADH dehydrogenase genes: *nad1*–*nad6* and *nad4l*; 3 cytochrome c oxidase genes: *cox1*–*cox3*; 1 cytochrome b gene: *cob*; and 2 ATP synthase genes: *atp6* and *atp8*), as well as 22 tRNA genes and 2 rRNA genes (12S and 16S) (Table S2).

**Figure 5:**
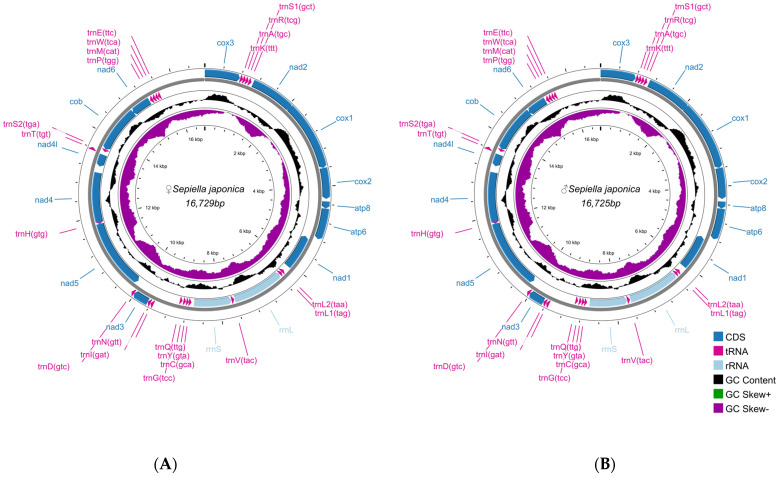
Mitochondrial map of female and male *S. japonica*. (**A**) Female. (**B**) Male. The first and second circles display the CDSs, rRNAs, and tRNAs located on both the positive and negative strands. The third circle represents the GC content. The inward-facing peaks of the circle indicate that the GC content was lower than the average value of the entire genome, and the outward-facing peaks indicate increases in the GC content relative to the average value of the genome. The fourth circle shows the GC-skew value.

The majority strand contained the *trnT*, *trnK*, *trnA*, *trnR*, *trnS1*, *trnN*, *trnI*, and *trnD* genes, along with the *cox1*, *cox2*, *cox3*, *nad2*, *nad3*, *atp6*, and *atp8* genes; the minority strand contained the rest of the mitochondrial genes. The same numbers of transfer RNA (tRNA) and ribosomal RNA (rRNA) genes were identified in both sexes (each with 22 tRNA genes and 2 rRNA genes) (Figure 5).

### 3.4. Phylogenetic Relationships of S. japonica Based on Mitochondrial Genome

**Figure 6:**
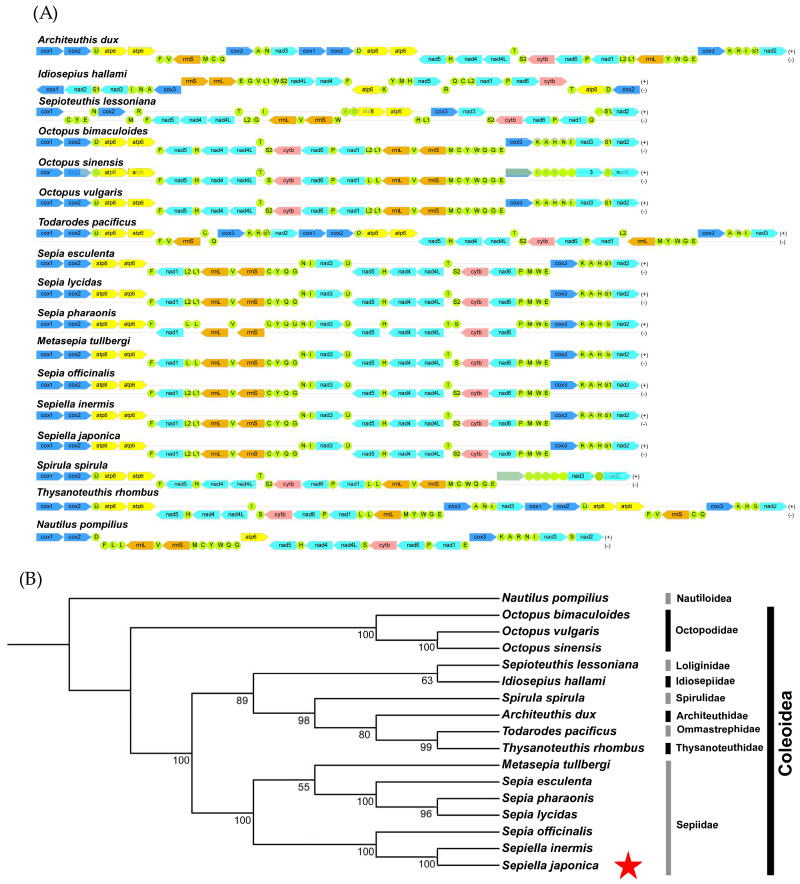
The phylogenetic tree reconstructed from the nucleotide sequences of thirteen PCGs using IQ-TREE in PhyloSuite. (**A**) The gene orders of the concatenated nucleotide sequences of protein-coding genes (PCGs) for different species. (**B**) The phylogenetic tree of cephalopod species based on mitochondrial protein-coding genes. Clade groupings indicate family taxonomy. Numbers on the branches represent bootstrap values (red star indicates the study species).

The phylogenetic position of *S. japonica* was determined using the complete mitochondrial genomes of 17 representative cephalopod species from the families Sepiidae, Octopodidae, Loliginidae, Idiosepiidae, Spirulidae, Architeuthidae, Ommastrephidae, and Thysanoteuthidae. *N. pompilius* was selected as an outgroup to root the phylogenetic tree. The tree was constructed from concatenated nucleotide sequences of all 13 mitochondrial protein-coding genes (PCGs) (Figure 6A). *S. japonica* clustered most closely with its congener *S. inermis*, and these two species formed a distinct clade that further grouped with *S. officinalis*, indicating a close evolutionary relationship between the genera *Sepiella* and *Sepia* (Figure 6B). *M. tullbergi* and three species of *Sepia* formed another clade, and these two clades together constituted the family Sepiidae. Except for the basal node (bootstrap = 55), all internal nodes within Sepiidae received full bootstrap support (bootstrap = 100), suggesting that the phylogenetic relationships among genera and species in this family are overall stable and well resolved [46]. Within other lineages, *O. bimaculoides*, *O. sinensis*, and *O. vulgaris* formed a single branch with maximal support (bootstrap = 100), supporting the monophyly of these three *Octopus* species. *T. pacificus* and *T. rhombus* grouped as sister branches (bootstrap = 99), indicating a close relationship between these taxa. In contrast, *S. lessoniana*, *I. hallami*, *S. spirula*, and *A. dux* formed a larger branch with lower support (bootstrap = 63), suggesting that the phylogenetic relationships among these taxa remain uncertain [47]. Overall, the topology of the phylogenetic tree was largely consistent with the traditional classification of cephalopods and clarified the evolutionary relationships of *S. japonica* with its congeners and closely related species, providing important evidence for future studies on the systematics and evolutionary history of the family Sepiidae.

### 3.5. Population Size Dynamics of S. japonica

**Figure 7:**
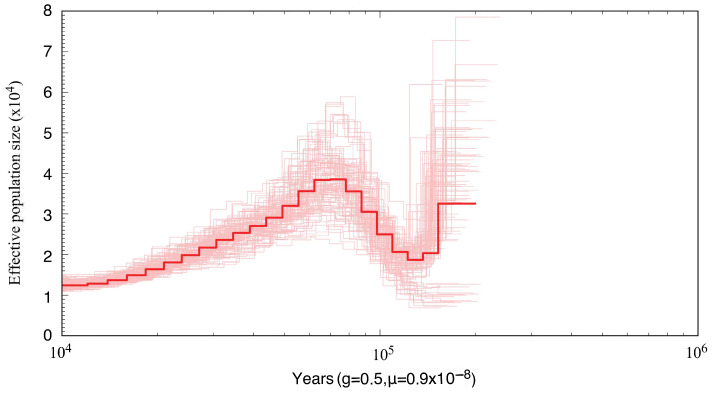
The demographic history of *S. japonica* in this study. The PSMC estimates based on the draft genome sequences of *S. japonica* revealed temporal changes in effective population size. The thin light-red lines represent 100 bootstrap replicates, and the thick red line indicates the median value.

The PSMC model was employed to infer the historical changes in the effective population size of *S. japonica* (Figure 7). The results showed that *S. japonica* experienced substantial demographic fluctuations and a bottleneck effect over the past ~300 Kya. Around 200 Kya, its effective population size remained at a moderate level (about 2–3 × 10^4^). A marked contraction then occurred, with the effective population size declining to below about 2 × 10^4^. Around 100 Kya, the population recovered and reached a peak (about 4 × 10^4^), followed by a continuous decline. During the Last Glacial Period (approximately ~70–15 Kya), the effective population size dropped to its minimum and showed no sign of recovery by about 10 Kya. The bootstrap analyses were consistent with the main curve within 100 Kya, indicating that the inference in this time range was robust.

## 4. Discussion

This study used NGS to conduct a genome survey analysis of female and male *S. japonica*, which yielded fundamental genomic information, including information on size, heterozygosity, and the content of repeats. Microsatellite motifs were also identified, and the complete mitochondrial genomes were assembled and annotated. Through comparative analysis, this study provided key insights into the basic genomic differences between female and male individuals of *S. japonica*. At present, only a few cephalopod species have undergone whole-genome sequencing, and systematic comparative analyses of female and male genomes remain scarce. For example, Li et al. combined a chromosome-level genome of 2.72 Gb of female *O. sinensis* using PacBio sequencing, Illumina paired-end sequencing, and Hi-C technology [48]. Similarly, Kim et al. assembled a 5.09 Gb genome for *Octopus minor*, providing valuable insights into the evolutionary adaptations of octopuses to mudflat environments [49]. Coffing et al. generated a 2.3 Gb genome assembly of *O. bimaculoides* by re-sequencing a single female individual using PacBio HiFi long-read sequencing, with Hi-C data used to anchor scaffolds to chromosomes [50]. Therefore, this study provides valuable data of great significance for the whole-genome assembly analysis of *S. japonica*. The genome sizes of female and male *S. japonica* were estimated to be 4311 Mb and 4215 Mb, respectively (corrected values), according to K-mer analysis. These values are slightly smaller than those reported for other related species, including *S. esculenta* (5.1 Gb, GCA_964036315.1), *Sepia bandensis* (5.9 Gb, GCA_037127315.1), *S. pharaonis* (4.8 Gb, GCA_903632075.3), and *S. lycidas* (5.2 Gb, GCA_963932145.1). This difference might stem from the lower content of repetitive elements, such as LINE and SINE transposable elements, in *S. japonica* compared with other *Sepiella* species. LINE elements comprise over 12% of the genome of *O. bimaculoides*, suggesting that they play a key role in genome expansion in cephalopods [9]. The lower abundance of transposable elements in *S. japonica* might contribute to the smaller size of their genome. Differences in genome size may be associated with reductions in the size of introns and intergenic regions. Cephalopod genomes are generally large and often range from 3 to 6 Gb; they have high proportions of repetitive sequences [9,51], which are potentially linked to their complex behavioral regulation, advanced nervous systems, and environmental adaptability. The observed variation in genome size among species likely reflects differences in the expansion of transposable elements, the accumulation of repeats, gene loss, or genome compaction over evolutionary time.

The estimated heterozygosity was 0.85% and 0.77% in females and males, respectively; repetitive sequences comprised 76.05% and 75.91% of the female and male genomes, respectively. Comparable genome survey analyses, all based on K-mer methods, have reported heterozygosity values ranging from 0.34% in *O. sinensis* [50] and 0.35% in *S. pharaonis* [5] to 1.64% in *Mytilus coruscus* [52]. These results indicate that the heterozygosity observed in *S. japonica* falls within the range reported for cephalopods and other marine mollusks.

The higher heterozygosity in females compared with males suggests that multiple factors such as reproductive strategies, environmental adaptability, and selection pressures have affected the genome of female *S. japonica*. For example, a genomic analysis of *Hapalogenys analis* revealed that females have higher heterozygosity (0.58%) than males (0.23%), indicating that females may experience stronger selection associated with mate choice and intraspecific competition compared with males [53]. Coffing et al. proposed that sex determination mechanisms affect genomic heterozygosity in the California two-spot octopus (*O. bimaculoides*); males have two copies of chromosome 17 (ZZ), and females have a single copy (ZO) [50]. These findings suggest that sex plays an important role in the formation and maintenance of genomic diversity in cephalopods. However, the causes of sex-related differences in heterozygosity are complex and might vary across taxa; additional studies are needed to further clarify this possibility. The somatic chromosome number of *S. japonica* is 2n = 92, with no morphologically distinct sex chromosomes observed [54]. This suggests that genomic differences between males and females likely originate from sequence-level variation rather than chromosomal dimorphism. Obtaining sex-specific karyotypic and sex chromosome data in the future will be essential to further elucidate these genomic differences.

The GC content was 35.16% and 35.27% in female and male *S. japonica*, respectively. The GC content is an important genomic characteristic associated with randomness in the genome and the density of functional genes [55,56]. The GC content in closely related species such as *S. esculenta* (33.5%), *S. bandensis* (34.5%), *S. pharaonis* (33.0%), and *S. lycidas* (33.0%) was similar to that in *S. japonica* in this study. This may reflect the evolutionary conservation of genome composition within cephalopods. The genome of *S. japonica* is moderately sized, has a high proportion of repetitive sequences, and has a relatively high GC content, which is consistent with the typical characteristics of cephalopod genomes, including a high repeat content and low coding density. Subsequently, comprehensive whole-genome sequencing research can be conducted.

Mononucleotide repeats were the most abundant in the female (38.59%) and male (38.92%) genomes, and hexanucleotide repeats were the least common. Generally, the number of SSRs decreases with the length of the repeating unit [53]. In this study, the proportion of tetranucleotide repeats was higher than the proportion of trinucleotide repeats in both female and male genomes. This may stem from the ease with which tetranucleotide repeats can be amplified and accumulate in non-coding regions [57]. The expansion and accumulation of tetranucleotide motifs may be facilitated by the high proportion of non-coding sequences. Overall, SSR types and frequencies were highly similar in female and male *S. japonica* genomes.

The whole mitochondrial genome of *S. japonica* was previously assembled by Yamashita et al. [58]. This study generated complete mitochondrial genome assemblies for both female and male *S. japonica*. A 551 bp non-coding region between *trnG* and *trnN* in the newly assembled sequences was identified as the mitochondrial control region; this region is essential for regulating mitochondrial DNA replication and transcription. Similar control regions have also been identified in closely related species (e.g., *S. lycidas*) [59]; this control region might be functionally significant and evolutionarily conserved in cephalopods. Compared with previous mitochondrial studies that have primarily focused on protein-coding regions, this study identifies and annotates the mitochondrial non-coding regions of *S. japonica*, providing preliminary insights into their potential roles in gene regulation and evolutionary adaptation. Furthermore, our analysis of the mitochondrial genome identified functionally significant genes and enhanced our understanding of the genomic structure of this species. Our findings provide novel molecular insights that will aid future studies of the evolution, reproduction, and systematics of *S. japonica* and its closely related species.

A substantial fall in sea level would have caused widespread exposure of continental shelves, a drastic reduction and fragmentation of shallow-marine habitats, and this process may have directly reduced the effective population size of *S. japonica* and promoted the differentiation of its population genetic structure [60,61,62]. As a species inhabiting nearshore environments [2], its population size is strongly influenced by large sea-level fluctuations [63]. PSMC analysis indicates that this species underwent a prolonged population decline beginning around 60 Kya and remained at a low effective population size until about ~10 Kya. This long-term reduction was likely closely associated with the cold climate and marked sea-level oscillations of the Last Glacial Period (approximately ~70–15 Kya) [60]. By altering habitat availability and connectivity, these environmental drivers probably governed the historical population decline of *S. japonica* and shaped its present genetic structure. Future studies integrating chromosome-level genome assembly with population resequencing will further elucidate its genetic structure and evolutionary history.

## 5. Conclusions

We conducted the first genome survey analysis of female and male *S. japonica*. We analyzed key genomic characteristics, including genome size, heterozygosity, GC content, and repeat sequences, and generated draft genome assemblies. The genome sizes of the females and males were 4310 Mb and 4215 Mb, respectively. The heterozygosity of the female genome was slightly higher than that of the male genome; however, the number of SSR markers was higher in the male genome than in the female genome. Overall, the differences in genomic characteristics between males and females were relatively small. Phylogenetic analysis based on the complete mitochondrial genome confirmed the taxonomic position of *S. japonica* within the family Sepiidae, showing its closest relationships with *S. inermis* and its placement within a stable clade including *S. officinalis*. PSMC analysis indicated that this species underwent a prolonged population decline during the Last Glacial Period (approximately ~70–15 Kya), likely driven by cold climate and pronounced sea-level fluctuations. Overall, our results reveal that *S. japonica* is a diploid species and highlight the challenges posed by its high heterozygosity and repetitive sequences for genome assembly. These results provide genomic resources for high-quality whole-genome assembly and comparative analysis of female and male *S. japonica*, and offer scientific references for subsequent studies on the genetic basis and evolutionary mechanisms of sex determination and differentiation, as well as for molecular breeding and germplasm resource conservation.

## Figures and Tables

**Table 1:** NCBI accession of mitogenomes of 17 species used in this study.

Species	Accession	Length (bp)	Family
*Nautilus pompilius*	NC_035715.1	15,693	Nautilidae
*Octopus bimaculoides*	NC_029723.1	15,733	Octopodidae
*Octopus vulgaris*	NC_006353.1	15,744	Octopodidae
*Sepioteuthis lessoniana*	NC_007894.1	16,631	Loliginidae
*Idiosepius hallami*	KF647895.1	16,183	Idiosepiidae
*Spirula spirula*	NC_034682.1	15,472	Spirulidae
*Architeuthis dux*	NC_011581.1	20,331	Architeuthidae
*Todarodes pacificus*	NC_006354.1	20,254	Ommastrephidae
*Thysanoteuthis rhombus*	NC_058301.1	20,545	Thysanoteuthidae
*Metasepia tullbergi*	MT974497.1	16,182	Sepiidae
*Sepia esculenta*	NC_009690.1	16,199	Sepiidae
*Sepia pharaonis*	NC_021146.1	16,208	Sepiidae
*Sepia lycidas*	NC_022468.1	16,244	Sepiidae
*Sepia officinalis*	NC_007895.1	16,163	Sepiidae
*Sepiella inermis*	NC_022693.1	16,191	Sepiidae
*Octopus sinensis*	NC_052881.1	15,737	Octopodidae
*Sepiella japonica*	PX243620	16,729	Sepiidae

**Table 2:** Statistics of sequencing data of female and male *S. japonica*.

Library Name	Type	Read Number	Base Count (Gb)	Read Length (bp)	Q20 (%)	Q30 (%)	GC Content (%)
C1M-J-1	raw	392,466,944	58.87	150	99.45	97.99	35.30
	dedup	386,993,714	57.30	148	99.45	97.99	35.12
C1M-J-2	raw	1,533,736,568	230.06	150	99.45	98.01	35.38
	dedup	1,459,025,604	216.77	148	99.45	98.01	35.24
C1M-J-3	raw	1,519,472,688	227.92	150	99.43	97.92	35.26
	dedup	1,446,429,734	214.51	148	99.43	97.92	35.10
Total	raw	3,445,676,200	516.85	150	99.44	97.97	35.32
	dedup	3,292,449,052	488.58	148	99.44	97.97	35.16
X1M-J-1	raw	351,953,724	52.79	150	99.50	98.17	35.57
	dedup	347,458,202	51.34	147	99.50	98.17	35.35
X1M-J-2	raw	1,457,509,058	218.63	150	99.44	97.96	35.46
	dedup	1,388,606,834	205.78	148	99.44	97.96	35.27
X1M-J-3	raw	1,648,035,630	247.21	150	99.40	97.83	35.45
	dedup	1,562,234,556	231.34	148	99.40	97.83	35.26
Total	raw	3,457,498,412	518.63	150	99.43	97.92	35.47
	dedup	3,298,299,592	488.46	148	99.43	97.92	35.27

Read number: Number of sequencing reads; Base count (Gb): Total bases sequenced in gigabases; Read length (bp): Average read length in base pairs; GC content (%): Percentage of guanine (G) and cytosine (C) bases; dedup: duplicate-removed data; C1M-J-1: Female-1; X1M-J-1: Male-1.

**Table 3:** Data statistics and analysis of 17-mer sequences in female and male *S. japonica*.

Sample	K-mer Number	K-mer Depth	Genome Size (bp)	Revised Genome Size (bp)	Heterozygous Ratio (%)	Repeat (%)
Female	435,897,116,926	102	4,317,320,000	4,310,509,074	0.85	76.05
Male	435,678,168,857	102	4,222,250,000	4,215,254,265	0.77	75.91

**Table 4:** Assembly statistics of the draft genomes of female and male *S. japonica*.

		Total Length (bp)	Total Number	Max Length (bp)	N50 Length (bp)	N90 Length (bp)	GC Content (%)
Female	contig	4,197,030,785	21,378,004	23,523	508	57	34.15
Male	contig	4,206,358,660	21,355,316	23,780	511	57	34.15

**Table 5:** Smudgeplot analysis results of female and male *S. japonica*.

	Peak	K-mers	K-mers [Proportion]	Summit B/(A + B)	Summit A + B
Female	AB	63,723,397	0.54	0.48	98.06
	AABB	34,490,621	0.29	0.48	199.5
	AAB	20,076,357	0.17	0.34	148.78
Male	AB	62,713,232	0.54	0.48	92.96
	AABB	34,793,915	0.30	0.49	205.99
	AAB	18,889,638	0.16	0.34	143.19

**Table 6:** Microsatellite motif types detected in female and male *S. japonica*.

	Female	Male
Total number of sequences examined	21,378,004	21,355,316
Total size of examined sequences (bp)	4,197,030,785	4,206,358,660
Total number of identified SSRs	4,347,973	4,322,277
Number of SSR containing sequences	3,175,080	3,152,792
Number of sequences containing more than 1 SSR	789,477	785,798
Number of SSRs present in compound formation	666,189	658,486

## Data Availability

The original contributions presented in this study are included in the article/Supplementary Materials. Further inquiries can be directed to the corresponding author.

## Supplementary Materials

The following supporting information can be downloaded at https://www.mdpi.com/article/10.3390/genes16101215/s1; Table S1: Comparison results of female and male *S. japonica* genome survey sequencing data in NT database; Table S2: Mitochondrial genome characteristics of *S. japonica*.
