# Supplementary material for: The Genome Survey Analysis of Female and Male Sepiella japonica
Source: Genes (Basel). 2025 Oct 15;16(10):1215. doi: 10.3390/genes16101215 (PMC12564688; doi:10.3390/genes16101215)
Supplement: Supplementary file 1 [file genes-16-01215-s001.zip › genes-3836425-supplementary.pdf]

Table S1 Comparison results of female and male *S. japonica* genome survey sequencing data in NT database

Genus	Kingdom	Percentage of hits (%)						Mean (%)
		C1M-J-1	C1M-J-2	C1M-J-3	X1M-J-1	X1M-J-2	X1M-J-3	
Sepia	Metazoa	23.55	24.16	19.71	19.37	22.61	22.96	22.06
Octopus	Metazoa	11.39	10.86	11.11	10.02	12.40	13.89	11.61
Sepiella	Metazoa	12.36	10.30	13.26	11.35	8.88	10.19	11.06
Papaver	Viridiplantae	8.11	7.87	6.99	7.35	6.87	7.96	7.53
Macrobrachium	Metazoa	2.51	4.31	4.12	3.84	4.19	2.22	3.53
Marthasterias	Metazoa	2.51	1.50	2.15	2.67	1.84	1.67	2.06
Sphaeramia	Metazoa	1.74	0.75	2.87	1.84	2.01	0.93	1.69
Ophonus	Metazoa	1.16	2.06	1.61	1.84	1.68	0.93	1.55
Odontocerum	Metazoa	1.93	1.50	0.72	0.83	1.84	2.41	1.54
Syzygium	Viridiplantae	2.70	1.69	0.72	1.50	1.01	1.48	1.52
Neoitamus	Metazoa	1.93	1.12	1.79	1.34	0.50	0.93	1.27
Sacculina	Metazoa	1.16	1.31	1.25	1.17	1.01	1.11	1.17
Crepidodera	Metazoa	0.77	0.56	1.79	0.67	0.50	1.48	0.96
Caligus	Metazoa	1.16	1.12	0.36	1.00	1.34	0.74	0.95
Architeuthis	Metazoa	0.58	0.56	0.54	1.00	1.01	1.67	0.89
Scyliorhinus	Metazoa	0.58	0.75	1.25	0.00	0.84	0.74	0.69
Thalassophryne	Metazoa	1.35	0.56	0.36	0.50	1.01	0.37	0.69
Harmonia	Metazoa	0.58	0.56	0.72	0.50	0.00	0.00	0.39
Ocypus	Metazoa	0.58	0.56	0.36	0.00	0.00	0.56	0.34

Table S2 Mitochondrial genome characteristics of *S. japonica*

Gene	Position (Female)		Position (Male)		Size (bp)		strand
	From	To	From	To	Female	Male	
cox3	1	780	1	780	780	780	majority
trnK	787	853	787	853	67	67	majority
trnA	852	917	852	917	66	66	majority
trnR	917	981	917	981	65	65	majority
trnS1	985	1051	985	1051	67	67	majority
nad2	1052	2089	1052	2089	1038	1038	majority
cox1	2061	3593	2061	3593	1533	1533	majority
cox2	3596	4279	3596	4279	684	684	majority
atp8	4335	4490	4335	4490	156	156	majority
atp6	4493	5185	4493	5185	693	693	majority
trnF	5211	5276	5211	5276	66	66	minority
nad1	5276	6213	5276	6213	938	938	minority
trnL2	6214	6284	6214	6284	71	71	minority

Continued table S2 Mitochondrial genome characteristics of *S. japonica*

Gene	Position (Female)		Position (Male)		Size (bp)		strand
	From	To	From	To	Female	Male	
trnL1	6287	6355	6287	6355	69	69	minority
rrnL	6356	7638	6356	7637	1283	1282	minority
trnV	7638	7707	7637	7706	70	70	minority
rrnS	7710	8692	7709	8689	983	981	minority
trnC	8693	8758	8690	8756	66	67	minority
trnY	8757	8821	8755	8819	65	65	minority
trnQ	8828	8893	8826	8891	66	66	minority
trnG	8915	8980	8913	8978	66	66	minority
Control region	8981	9531	8979	9529	551	551	majority
trnN	9532	9599	9530	9597	68	68	majority
trnI	9602	9668	9600	9666	67	67	majority
nad3	9969	10022	9667	10020	54	354	majority
trnD	10021	10085	10019	10083	65	65	majority
nad5	10113	11822	10111	11820	1710	1710	minority
trnH	11823	11890	11821	11888	68	68	minority
nad4	11844	13250	11842	13248	1407	1407	minority
nad4l	13247	13543	13245	13541	297	297	minority
trnT	13558	13623	13556	13621	66	66	majority
trnS2	13624	13687	13622	13685	64	64	minority
cob	13687	14826	13685	14824	1140	1140	minority
nad6	14819	15331	14817	15329	513	513	minority
trnP	15333	15404	15331	15402	72	72	minority
trnM	15405	15475	15404	15474	71	71	minority
trnW	15478	15544	15477	15543	67	67	minority
trnE	15547	15613	15546	15612	67	67	minority
Control region	15614	16725	15613	16729	1112	1117	majority
